# Key Hub and Bottleneck Genes Differentiate the Macrophage Response to Virulent and Attenuated *Mycobacterium bovis*

**DOI:** 10.3389/fimmu.2014.00422

**Published:** 2014-10-01

**Authors:** Kate E. Killick, David A. Magee, Stephen D. E. Park, Maria Taraktsoglou, John A. Browne, Kevin M. Conlon, Nicolas C. Nalpas, Eamonn Gormley, Stephen V. Gordon, David E. MacHugh, Karsten Hokamp

**Affiliations:** ^1^Animal Genomics Laboratory, UCD School of Agriculture and Food Science, University College Dublin, Dublin, Ireland; ^2^Systems Biology Ireland, UCD Conway Institute of Biomolecular and Biomedical Research, University College Dublin, Dublin, Ireland; ^3^IdentiGEN Ltd., Dublin, Ireland; ^4^Biological Agents Unit, Health and Safety Executive, Leeds, UK; ^5^UCD School of Veterinary Medicine, University College Dublin, Dublin, Ireland; ^6^Science Foundation Ireland (SFI), Dublin, Ireland; ^7^Tuberculosis Diagnostics and Immunology Research Centre, UCD School of Veterinary Medicine, University College Dublin, Dublin, Ireland; ^8^UCD Conway Institute of Biomolecular and Biomedical Research, University College Dublin, Dublin, Ireland; ^9^Smurfit Institute of Genetics, Trinity College, Dublin, Ireland

**Keywords:** *Mycobacterium bovis*, tuberculosis, BCG, cattle, macrophage, network, gene interaction network

## Abstract

*Mycobacterium bovis* is an intracellular pathogen that causes tuberculosis in cattle. Following infection, the pathogen resides and persists inside host macrophages by subverting host immune responses via a diverse range of mechanisms. Here, a high-density bovine microarray platform was used to examine the bovine monocyte-derived macrophage transcriptome response to *M. bovis* infection relative to infection with the attenuated vaccine strain, *M. bovis* Bacille Calmette–Guérin. Differentially expressed genes were identified (adjusted *P*-value ≤0.01) and interaction networks generated across an infection time course of 2, 6, and 24 h. The largest number of biological interactions was observed in the 24-h network, which exhibited scale-free network properties. The 24-h network featured a small number of key hub and bottleneck gene nodes, including *IKBKE, MYC, NFKB1*, and *EGR1* that differentiated the macrophage response to virulent and attenuated *M. bovis* strains, possibly via the modulation of host cell death mechanisms. These hub and bottleneck genes represent possible targets for immuno-modulation of host macrophages by virulent mycobacterial species that enable their survival within a hostile environment.

## Introduction

*Mycobacterium bovis*, the causative agent of bovine tuberculosis (BTB) is a facultative pathogen that has a genome sequence 99.95% identical to that of *M. tuberculosis*, the etiological agent responsible for human tuberculosis (TB) (1). The pathogenesis of BTB is broadly similar to that of human TB and many of the characteristics of *M. tuberculosis* infection are thought to apply to *M. bovis* infection in cattle (2, 3). Both pathogens enter the host principally via inhalation of infected aerosol droplets, where they are phagocytosed by alveolar macrophages and dendritic cells. Pathogenic mycobacteria have evolved mechanisms for subverting the host immune response, including prevention of phagosome maturation and subsequent lysosomal delivery (4), enabling survival and replication within the host macrophage following phagocytosis (5). Infected alveolar macrophages can then remain within the central core of granulomas during latent infection or disseminate to draining lymph nodes and to other host organs during active infection (6, 7).

*Mycobacterium bovis* Bacille Calmette–Guérin (BCG) is an attenuated strain of *M. bovis* that has been used as a live vaccine for TB control for almost a century. It is estimated that over 100 million people are immunized with BCG annually, making it the most widely used vaccine in human populations (8). All BCG strains available today are derived from a virulent *M. bovis* strain that was isolated at the turn of the twentieth century from the milk of an infected cow with tubercular mastitis (9). This strain, named “lait Nocard,” was transferred to the Institut Pasteur in Lille in 1901, where it was cultured on glycerol-soaked potato slices supplemented with ox bile by Albert Calmette and Camille Guérin as part of their studies of TB pathogenesis. Serial passage of the lait Nocard strain on this culture medium resulted in the isolation of a strain, in 1908, that displayed reduced virulence in guinea pigs and calves (9). Over the next 13 years, Calmette and Guérin performed 230 *in vitro* serial passages of this partially attenuated *M. bovis* strain, eventually leading to the first successful vaccination of a human infant with a fully attenuated BCG isolate in July 1921 (10, 11).

All currently existing BCG substrains originate from the initial attenuated strain developed by Calmette and Guérin during this 13-year period. It was during this time that BCG underwent the first of two attenuation phases. Early studies identified a region of difference 1 (RD1) that is deleted from all BCG strains (12, 13) and was likely lost during the initial attenuation phase between 1908 and 1921. RD1 encodes, among other genes, the early secretory antigen target (ESAT-6) protein secretion system (ESX-1), which is present in virulent strains of *M. bovis* and *M. tuberculosis* (14, 15). A second attenuation phase occurred after 1924 due to dissemination of the BCG vaccine from the Institut Pasteur to other countries (13). During this time further genomic deletions occurred, including the RD2 deletion (8, 9).

Virulent *M. bovis* and attenuated BCG differ in their ability to generate disease. Infection with *M. bovis* can lead to subversion of the host immune response and formation of tuberculous granulomas with characteristic BTB pathology (16). In contrast, the BCG vaccine strain does not generally cause progressive infection in immune-competent hosts, but can induce a protective pro-inflammatory T_H_1 response via activation of interferon-gamma (IFN-γ) secreting CD4^+^ T-cells (17); however, the precise mechanisms of how protection is afforded against virulent challenge remain unclear.

Despite decades of research, the host innate immune mechanisms elicited following infection with virulent *M. bovis* relative to attenuated BCG remain largely unknown. To our knowledge, a systems biology approach to understanding innate immune responses to virulent *M. bovis* and attenuated BCG has not previously been described. Systems analysis of transcriptomics data generated from host macrophages infected with the two strains enables reconstruction of dynamic molecular interaction networks that capture the differential innate immune responses to virulent *M. bovis* and attenuated BCG (18). Network features such as hub and bottleneck genes – a consequence of scale-free behavior – can also be defined, cataloged, and further explored to identify novel biological attributes relevant to the pathogenesis of mycobacterial infections.

Previous studies have demonstrated that the bovine monocyte-derived macrophage (MDM) is a useful model cell type for understanding the early host immunogenetic response to *M. bovis* and *M. bovis*-derived antigens (19–22). In the present study, the Affymetrix^®^ GeneChip^®^ Bovine Genome Array was used to examine the transcriptome of MDM from seven age-matched Holstein-Friesian female cattle infected *in vitro* with *M. bovis* and BCG across an infection time course of 2, 6, and 24 h. Differentially expressed (DE) genes were identified using established methods and a list of interactions were generated using the InnateDB resource (23) and visualized as interaction networks with the Cytoscape software package (24).

## Materials and Methods

### Ethics statement

All animal procedures were carried out according to the provisions of the Cruelty to Animals Act (Irish Department of Health and Children license number B100/3939) and ethical approval for the study was obtained from the UCD Animal Ethics Committee (protocol number AREC-P-07-25-MacHugh).

### Animals

Seven age-matched (3-year-old) unrelated Holstein-Friesian females were used in this study. All animals were maintained under uniform housing conditions and nutritional regimens at the UCD Lyons Research Farm (Newcastle, County Kildare, Ireland). The animals were selected from an experimental herd without a recent history of BTB infection and all animals tested negative for the single intradermal tuberculin test (SICTT). These cattle were also negative for infection with *Brucella abortus, M. avium* subsp. *paratuberculosis, Salmonella Typhimurium*, bovine herpesvirus 1 (BHV-1), and bovine viral diarrhea (BVD) virus.

### Monocyte extraction and culture of MDM

Monocyte extraction and culture of bovine MDM was performed as described by us previously (20). Briefly, for monocyte isolation, 300 ml of whole blood was collected in acid citrate dextrose buffer (Sigma-Aldrich Ireland Ltd., Dublin, Ireland) in sterile bottles. Blood was layered onto Accuspin™ tubes containing Histopaque^®^ 1077 (Sigma-Aldrich Ireland Ltd., Dublin, Ireland), and following density gradient centrifugation (500 g for 20 min) performed at room temperature, peripheral blood mononuclear cells (PBMC) were collected. Contaminating red blood cells (RBC) were removed following resuspension and subsequent incubation of the PBMC in RBC lysis buffer (10 mM KHCO_3_, 150 mM NH_4_Cl, 0.1 mM EDTA pH 8.0) for 5 min at room temperature. After incubation, PBMC were washed twice with sterile phosphate-buffered saline (PBS; Invitrogen™, Life Technologies Corporation, Paisley, UK) before resuspending cells in PBS containing 1% bovine serum albumin (BSA; Sigma-Aldrich Ireland Ltd., Dublin, Ireland). Monocytes were then isolated using the MACS^®^ protocol and MACSH MicroBeads conjugated to mouse anti-human CD14 antibodies (Miltenyi Biotec Ltd., Surrey, UK), which has been shown to be cross-reactive with bovine monocytes (25). The MACS^®^ protocol was performed according to the manufacturer’s instructions.

The identity and purity of monocytes was confirmed by flow cytometry using an anti-CD14 fluorescein-labeled antibody (data not shown). This method has been previously shown by us to yield a purity of CD14^+^ cells ≥99% (19). Purified monocytes were seeded at 1 × 10^6^ per well on 24-well tissue culture plates in RPMI 1640 medium (Invitrogen™, Life Technologies Corporation, Paisley, UK) containing 15% heat inactivated fetal calf serum (FCS; Sigma-Aldrich Ireland Ltd., Dublin, Ireland), 1% non-essential amino acids (NEAA; Sigma-Aldrich Ireland Ltd., Dublin, Ireland), gentamicin (5 mg/ml; Sigma-Aldrich Ireland Ltd., Dublin, Ireland), and incubated at 37°C, 5% CO_2_. Following 24 h incubation (day one), the media was replaced with 1 ml fresh antibiotic-containing media to remove any non-adhered cells. On day three, media was replaced with 1 ml antibiotic-free culture media (RPMI 1640 medium containing 15% heat inactivated FCS and 1% NEAA only). To ensure that the same number of MDM were subjected to mycobacterial challenge, cells were dissociated on day five using 1× non-enzymatic cell dissociation solution (Sigma-Aldrich Ireland Ltd., Dublin, Ireland), counted, and then re-seeded at 2 × 10^5^ cells per well in 24-well tissue culture plates (Sarstedt Ltd., County Wexford, Ireland) using antibiotic-free culture media. By day eight, 80–100% confluent monolayers of MDM were generated that displayed the characteristic macrophage morphology as confirmed by Giemsa staining (data not shown). On day eight, MDM were used for the *in vitro* challenge experiments with *M. bovis* and *M. bovis* BCG.

### Culture of *M. bovis* and BCG and infection of bovine MDM

The culturing of *M. bovis* (strain M2137; spoligotype SB0142) has been described by us previously (20). Culturing of BCG (Pasteur strain) was performed in a Biosafety Containment Level 3 (CL3) laboratory and conformed to the national guidelines on the use of Hazard Group 3 infectious organisms. BCG stocks for the *in vitro* MDM infection experiments were cultured in Middlebrook 7H9 medium (Difco™, Becton, Dickinson Ltd., Oxford, UK) containing 10% (vol/vol) BBL™ Middlebrook OADC Enrichment (Difco™, Becton, Dickinson Ltd., Oxford, UK), 0.05% Tween 80 (Sigma-Aldrich, Dublin, Ireland), and 0.50% (weight/vol) glycerol (Sigma-Aldrich Ltd., Dublin, Ireland) at 37°C. Bacterial cultures were grown to mid-logarithmic phase as determined by spectrophotometric analysis prior to the challenge experiments using the purified bovine MDM. Colony-forming unit (cfu) counting was performed using Middlebrook 7H11 medium (Difco™, Becton-Dickinson Ltd., Dublin, Ireland) containing 10% (vol/vol) Middlebrook (ADC) enrichment (Difco™, Becton-Dickinson Ltd., Dublin, Ireland) and 0.50% (vol/vol) glycerol (Sigma-Aldrich, Dublin, Ireland).

Monocyte-derived macrophage infections with BCG were performed as previously described for MDM infections with *M. bovis* (20). In brief, MDM (seeded at 2 × 10^5^ cells per well) were challenged with BCG (4 × 10^5^ cells per well) prepared in antibiotic-free culture media (RPMI 1640 medium containing 15% heat inactivated FCS and 1% NEAA only). BCG cell counts were performed using a Petroff Hausser chamber (Fisher Scientific Ltd., Dublin, Ireland) following sterile filtering to prevent clumping using a 5 μm filter (Millipore Ireland Ltd., County Cork, Ireland). BCG cell numbers were then adjusted and 4 × 10^5^ bacilli were added to the appropriate wells giving a multiplicity of infection (MOI) of 2:1. Subsequent cfu for BCG counting also yielded a mean MOI of 2:1. Once challenged, MDM were incubated at 37°C, 5% CO_2_ for 2, 6, and 24 h.

### RNA extraction and microarray analysis

RNA extraction and microarray analysis has been described by us previously (20). Briefly, global gene expression was analyzed using the pan-genomic high-density Affymetrix^®^ GeneChip^®^ Bovine Genome Array (Affymetrix UK Ltd., High Wycombe, UK^1^). This array contains 24,072 probe sets representing over 23,000 gene transcripts and includes approximately 19,000 Uni-Gene clusters. cDNA labeling, hybridization, and scanning for the microarray experiments were performed by Almac Diagnostics Ltd. (Craigavon, Co. Armagh, Northern Ireland) using a one-cycle amplification/labeling protocol.

### Statistical analysis of microarray data

Affymetrix^®^ GeneChip^®^ Bovine Genome Array data were analyzed using BioConductor (26)^2^ contained within the R statistical package^3^. All raw data were normalized using the factor analysis for robust microarray summarization (FARMS) package. The FARMS package uses only perfect match (PM) probes and a quantile normalization procedure, providing both *P*-values and signal intensities (27). Normalized data were then further subjected to filtering for informative probes sets using the I/NI-calls package in R (28). This defines a probe set as being informative when many of its probes reflect the same change in mRNA concentration across arrays. DE genes for a paired comparison between *M. bovis* versus BCG infections at each time point were extracted using a moderated paired *t*-test within the Linear Models for Microarray Data (LIMMA) R package (29)^4^. Genes displaying differential expression were annotated using the Affymetrix^®^ bovine gene annotation. The Benjamini–Hochberg multiple-testing correction method (30) was applied to all DE genes to minimize the false discovery rate (FDR) and adjusted *P*-values for all DE genes were calculated. Only DE genes with an adjusted *P*-value of ≤0.01 were used for subsequent network reconstruction and analysis. All data are MIAME compliant and have been submitted to the NCBI gene expression omnibus (GEO) database with experiment series accession number GSE59774.

### Generation of InnateDB interaction lists

Affymetrix^®^ probe IDs for the DE gene lists at each time point were mapped to human Ensembl IDs, using the BioMart search tool on the Ensembl genome database^5^. Each of the three DE gene lists were uploaded separately to the InnateDB^6^ curated, interaction database (23), where a list of interactions between the uploaded molecules were generated. At the time of this analysis, the InnateDB resource contained over 195,000 experimentally determined interactions, of which more than 18,000 have been manually curated, allowing for a detailed systems-level analysis of the innate immune response. The interactions, for each time point, were then viewed as a reconstructed network using the Cytoscape software (24)^7^. Small clusters of nodes that did not connect to the main network were removed from the analysis.

### 24 h interaction network analysis

The 24-h interaction network was analyzed as an undirected, Markov, network. Statistical analysis of the network was performed using the *NetworkAnalyzer* plugin of Cytoscape (31). Hub nodes were identified by calculating the degree of connectivity (DOC) for each node in the network using the *Degree Sorted Circle Layout* in Cytoscape. DOC is a measure of the number of interactions a node has within the network. Nodes with the highest DOC are classed as hub nodes. Bottleneck nodes were identified by calculating the betweenness centrality index (BCI) for each node in the network, also using the *NetworkAnalyzer* plugin. BCI is a measure of how often a node appears on the shortest path between nodes in the network and is a reflection of the amount of control a node exerts over the interactions of other nodes in the network (32). A Spearman rank correlation between the DOC and log_2_ fold-change in gene expression (*M. bovis*-infected MDM relative to BCG-infected MDM) was performed in the SPSS statistics version 20 package (IBM, New York, NY, USA).

#### Combination of interactions and differentially expressed gene lists

Several Perl scripts were implemented to combine gene lists and interactions in various ways. One was used to calculate the numbers of genes that are DE and linked to a set of hub genes by a certain distance of edges/interactions, another one to extract expression values for interactors with bottleneck genes at all time points. All scripts are available through GitHub at: https://github.com/kkillick/Systems-biology-paper.git.

#### Gene ontology of 24 h network

Ingenuity^®^ systems pathway analysis (IPA) was used to identify gene ontology (GO) functional categories over-represented within the DE genes found in the 24-h interaction network. The Ingenuity^®^ knowledge base contains the largest database of manually curated and experimentally validated physical, transcriptional, and enzymatic molecular interactions. Furthermore, each interaction in the Ingenuity^®^ knowledge base is supported by previously published information. For IPA GO analysis, the Ingenuity^®^ knowledge base was used as a reference set and only genes within the 24-h interaction network were uploaded. IPA then performed an over-representation analysis that categorized the DE genes within the uploaded list into functional GO categories. Each GO category in IPA is ranked based on the number of DE genes falling into each functional group. Right-tailed Fisher’s exact tests were used to calculate an overlap *P*-value for each of the biological functions assigned to the list of DE genes.

#### Expression analysis of the interactors of the top hub and bottleneck genes

The top hub and bottleneck genes (*IKBKE, MYC, NFKB1*, and *EGR1*) were specified in InnateDB to generate lists of all human interactions. DE genes for a paired comparison of *M. bovis* versus BCG infections at each time point, using an adjusted *P*-value of ≤0.01, were extracted from the complete interaction lists and visualized using the Circos software package^8^(33). Interaction nodes colored in red indicate that the gene was up-regulated within the dataset, while green indicate that the gene was down-regulated.

#### Identification of scale-free and small-world properties within the 24-h network

If a network has a node degree distribution that can be fitted with a power law distribution, it can be a sign that the network has a scale-free architecture. Node degree distribution of the 24-h network was calculated using the *NetworkAnalyzer* plugin of cytoscape and a power law distribution was fitted using Microsoft Excel.

To determine whether a network displays small-world properties it must show $L\tilde{>}L_{\mathrm{random}}$ but $C\gg C_{\mathrm{random}}$. Where *L* is the characteristic path length, defined as the number of edges in the shortest path between two nodes, averaged over all pairs of nodes, and *C*, the clustering coefficient, is defined as follows: node *n* has *k_n_* max number of edges. *C_n_* denotes the fraction of these edges that actually exist, while *C* is defined at the average of *C_n_* over all *n* (34).

The R package igraph^9^ was used to generate 1,000 random networks, each with 716 nodes and 1,785 edges. The mean characteristic path length and the mean clustering coefficient were calculated across the 1,000 networks also using the average path length and transitivity functions of the igraph R package. All R code used here is available through GitHub at: https://github.com/kkillick/Systems-biology-paper.git.

### Real time quantitative reverse transcription (qRT)-PCR validation of microarray results

A panel of 12 genes that were DE across all three time points based on the microarray data was selected for microarray validation using real time quantitative reverse transcription PCR (qRT-PCR) analysis. The laboratory and statistical methods used to perform this analysis are described in Section “Real Time Quantitative Reverse Transcription (qRT)-PCR Validation of Microarray Results” in Supplementary Material, and Table S1 in Supplementary Material details the qRT-PCR primers used in this study.

## Results

### Increasing differential gene expression between *M. bovis*- versus BCG-infected MDM over time

**Figure 1:**
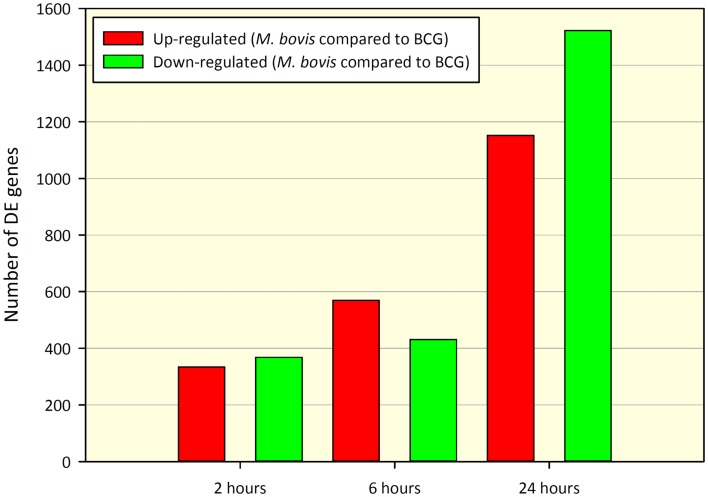
**Number of DE genes found for a paired comparison of virulent *M. bovis* versus attenuated BCG infections of bovine MDM (*n* = 7) across a time course of 2, 6, and 24 h post-infection**. DE gene numbers are reported here after filtering using an adjusted *P*-value of ≤ 0.01. Red bars indicate the number of genes displaying increased relative expression in *M. bovis* compared to BCG (up-regulated) and green bars indicate the number of genes displaying decreased relative expression in *M. bovis* compared to BCG (down-regulated).

Comparison of gene expression profiles between paired *M. bovis* versus BCG *in vitro* infections revealed 702 DE genes at 2 h and 1,000 and 2,674 DE genes at 6 and 24 h post-infection, respectively (adjusted *P*-value ≤0.01). Of the 702 DE genes found at the 2-h time point, 334 genes displayed increased expression in *M. bovis* infections relative to BCG infections (hereafter referred to as up-regulated), while 368 genes showed decreased expression in *M. bovis* infections relative to BCG infections (hereafter referred to as down-regulated). At the 6-h time point, 569 genes were up-regulated, with 431 genes displaying down-regulation, while at the 24-h time point 1,152 and 1,522 genes were up- and down-regulated, respectively. Tables S2–S4 in Supplementary Material contain DE gene results for the 2, 6, and 24 h post-infection time points, respectively. Figure 1 depicts the number of DE genes and the relative change in expression across the infection time course.

### InnateDB interaction analyses

InnateDB is an open-source database containing experimentally verified gene and protein-interactions involved in the human, mouse, and bovine innate immune response (23). As a larger number of curated interactions are available for human data, the DE genes detected here were mapped to corresponding human Ensembl gene IDs prior to analysis with InnateDB. In total, 362 DE genes at 2 h, 554 DE genes at 6 h, and 1,503 DE genes at 24 h mapped to human Ensembl gene IDs. These gene lists were examined separately using InnateDB to generate a catalog of interactions for each infection time point. At 2 h post-infection, 205 interactions were identified, while 413 and 1,785 interactions were detected for 6 and 24 h, respectively. As the 24-h time point generated the largest interaction network, it was chosen for detailed downstream analyses.

### 24 h interaction network reconstruction and analysis

Removal of small groups of nodes not linked to the main cluster left 615 nodes for analysis in the 24-h interaction network. These were connected via 1,670 edges. Two hundred eight of these nodes had self-interaction loops. The network diameter was 10 and the average shortest path length was 4.315. The *IKBKE, MYC, NFKB1, HDAC5*, and *TRAF2* genes were identified as the top hub nodes within the network. A degree of connectivity (DOC) of 57 was found for *IKBKE*, 57 for *MYC*, 55 for *NFKB1*, 52 for *HDAC5*, and 50 for *TRAF2*. The DOC for these five nodes was markedly higher than the mean number of connections found for nodes within the network of 3.857, suggesting that they are hub genes within the 24-h network. Also, for the 24-h interaction network, *IKBKE, MYC*, and *EGR1* displayed the highest betweenness centrality index (BCI) of 0.1661, 0.1360, and 0.1297, respectively (Figure S1 in Supplementary Material). This finding suggests that *IKBKE, MYC*, and *EGR1* are also key bottleneck nodes within the 24-h interaction network.

**Figure 2:**
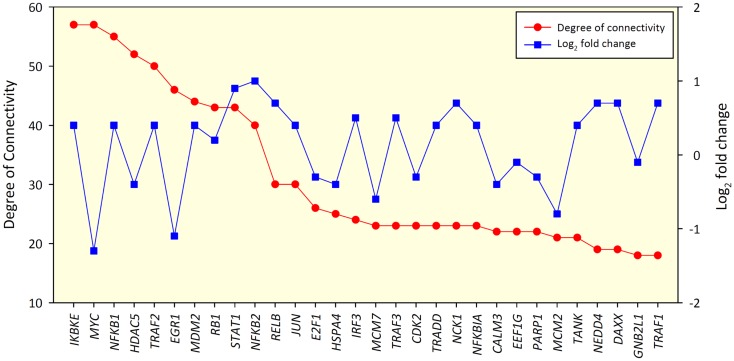
**Top 30 gene nodes in the 24-h interaction network that displayed the highest DOC (red line)**. The log_2_ fold-change values for these genes are also shown (blue line).

No significant correlation between the DOC and the log_2_ fold-change in gene expression for nodes was found, indicating that nodes with the greatest changes in expression between groups may not have the biggest influence on other nodes within the network (Spearman rho value 0.066, *P* = 0.108) (Figure 2). Figures S2 and S3 in Supplementary Material show the biological interaction networks for the 2 and 6-h infection time points, respectively.

### Scale-free and small-world properties of the *M. bovis* versus BCG 24 h interaction network

A power law distribution was fitted to the node degree distribution of the 24-h network, generating an *R*^2^ value of 0.867 (Figure S4 in Supplementary Material). This result suggests that the 24-h interaction network has a scale-free architecture, whereby many nodes have a small number of connections yet a small number of hub nodes have many connections. Scale-free networks tend to exhibit the small-world network property of high clustering and short average path lengths, $L\tilde{>}L_{\mathrm{random}}$ but $C\gg C_{\mathrm{random}}$, (where *L* is the characteristic path length and *C* is the clustering coefficient) (34). This was tested on the 24-h interaction network by comparing it to 1,000 random networks generated with the igraph *R* package using the same number of nodes and edges. The random networks yielded a mean *C* = 0.007, with a maximum *C* = 0.012 and *L* = 4.251. Conversely, for the observed 24 h interaction network, the *C* and *L* values were 0.112 and 4.315, respectively. With a *C* value 15.9-fold higher than the mean *C* value for the 1,000 random networks, and the *L* value only marginally higher, the 24-h interaction network exhibits small-world properties.

**Figure 3:**
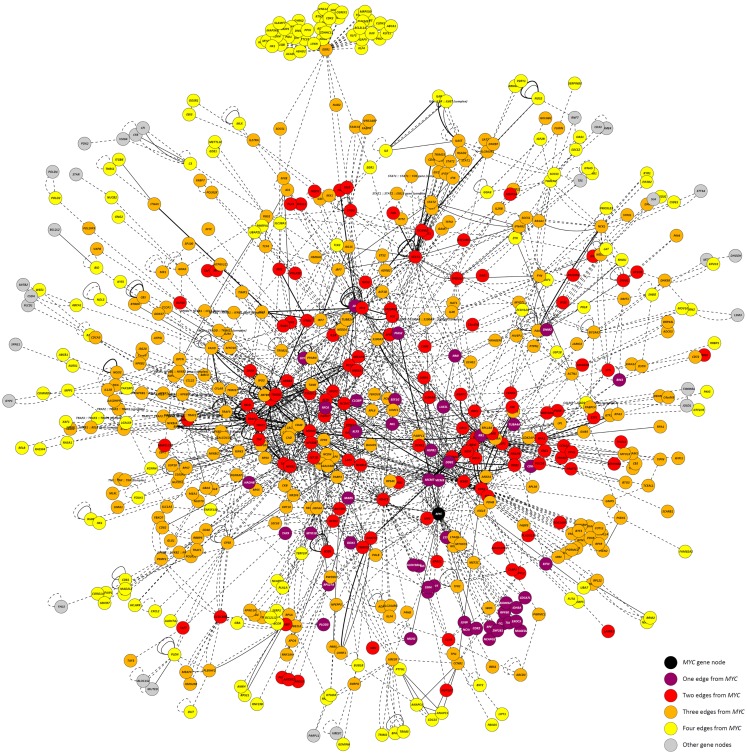
**The 24-h interaction network demonstrating scale-free network properties**. The main hub gene node, *MYC*, is shown in black. Gene nodes that are one edge/interaction from *MYC* are colored purple; gene nodes that are two edges/interactions from *MYC* are colored red; gene nodes that are three edges/interactions from *MYC* are colored orange; and gene nodes that are four edges/interactions from *MYC* are colored yellow. Gene nodes colored gray are greater than four edges/interactions from *MYC*.

**Figure 4:**
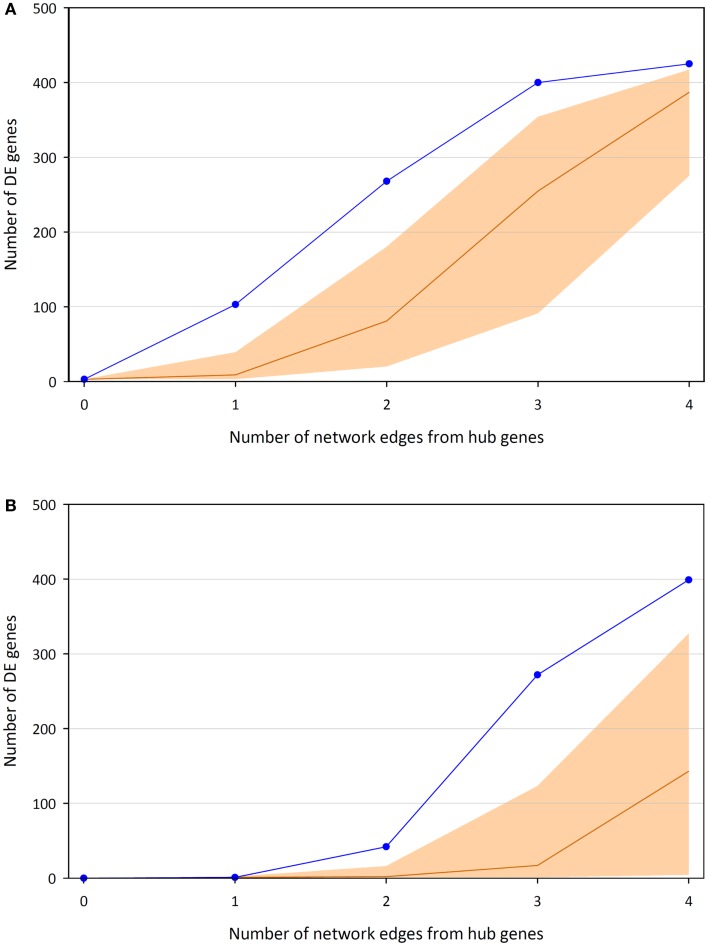
**Numbers of DE genes linked to at least one of the three key hub genes (*IKBKE, MYC*, and *NFKB1*) up to a certain distance in terms of edges/interactions**. **(A)** Union of genes linked to the hubs and median average of 100,000 permutations, including 95% of the distribution. **(B)** Intersection of genes linked to the hubs and median average of 100,000 permutations, including 95% of the distribution.

Highlighting the gene neighborhoods at increasing distances from a main hub node (*MYC*) in the 24-h interaction network demonstrates the scale-free architecture of the network. Figure 3 shows that almost all other nodes in the network (587 out of 615) are connected to *MYC* through less than five edges. The combined effect of the three main hub gene nodes (*IKBKE, MYC*, and *NFKB1*) is presented in Figure 4A. It shows that nearly all DE genes in the network (96%) are linked through a maximum of four edges to one of the three key hub genes. This is significantly higher than expected by chance (*P*-value < 0.01), based on 100,000 permutation tests. In addition, the intersection between the linked genes from all hubs rises quickly from less than 10% at a distance of two edges to nearly 90% at a distance of four edges (Figure 4B), indicating complex intertwinement and a capacity for rapid dissemination of signals throughout the network.

### Gene ontology of the *M. bovis* versus BCG 24 h interaction network

The total number of DE genes that could be mapped to molecules in the Ingenuity^®^ Knowledge Base was 507, out of the 615 genes in the uploaded dataset. GO analysis facilitates discovery of molecular functions that are enriched within a dataset. IPA GO analysis of molecules within the 24-h interaction network revealed *Cell death* to be a major over-represented functional category. Two hundred thirty-seven of the molecules within the 24-h network were involved in *Cell death* and an adjusted *P*-value of 4.18 × 10^−25^ was obtained for this category. Further inspection showed that the top 15 hub nodes and the top 15 bottleneck nodes were also involved in the *Cell death* GO category.

### Expression analysis of the interactors of the top hub and bottleneck genes

**Figure 5:**
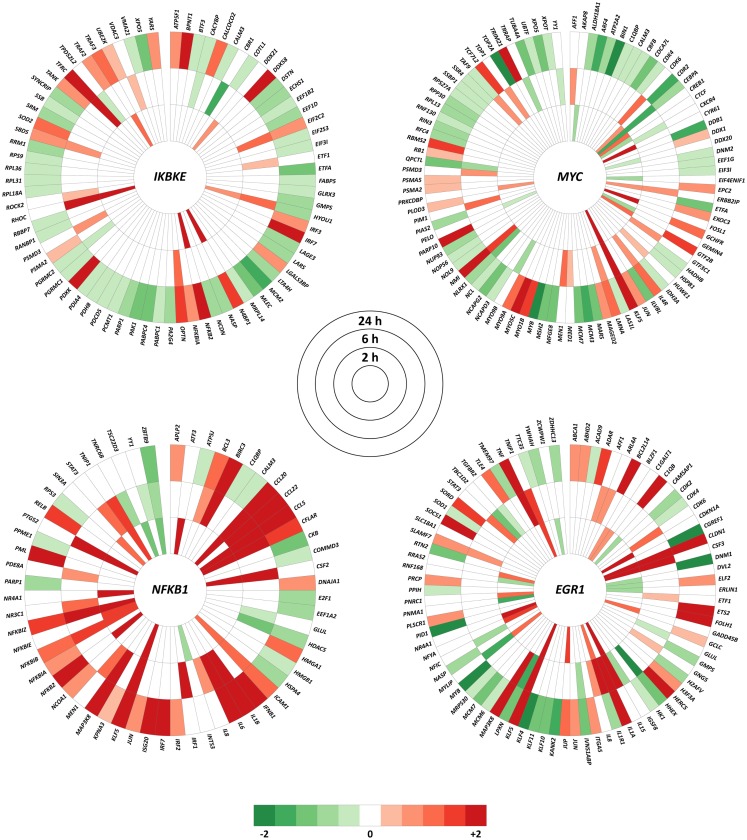
**All interactions within InnateDB for the *IKBKE, MYC*, and *NFKB1* hub genes plus the top bottleneck gene node (*EGR1*) that show significantly differential expression in at least one of the three time points using an adjusted *P*-value of ≤ 0.01**. Color intensity indicates the degree of up-regulation (red) or down-regulation (green) in the *M. bovis*- versus the BCG-infected bovine MDM (*n* = 7) at each time point.

Expression changes of the interactors with the top hub and bottleneck genes *IKBKE, NFKB1, MYC*, and *EGR1* were examined at each of the three time points. Using an adjusted *P*-value of ≤0.01, it was observed that the majority of the interactors were DE at 24 h for a paired comparison of *M. bovis* versus BCG infections. This is in contrast to the earlier time points where only small numbers of interactors exhibited significant expression changes (adjusted *P*-value ≤0.01). For example, the number of DE genes that interact with *IKBKE* increased from 6 to 12 between time points 2 and 6 h, but expanded to 68 at 24 h. These results suggest that differential regulatory activity at the *IKBKE* hub gene and its interactors increases over time in MDM infected with *M. bovis* relative to BCG. A similar outcome was observed for the *NFKB1* and *MYC* hub genes and the *EGR1* hub/bottleneck gene, where the equivalent numbers of DE genes for the 2, 6, and 24-h time points were 19, 27, and 42; 15, 22, and 72; and 23, 26 and 54, respectively (Figure 5).

### Real time quantitative reverse transcription PCR (qRT-PCR) validation of microarray results

The results from real time qRT-PCR validation of the microarray results are provided in Table S5 in Supplementary Material.

## Discussion

The ability of pathogenic mycobacteria to cause disease has been largely attributed to their capacity to survive and replicate in macrophages via a diverse range of mechanisms that subvert the host innate immune response (35). It is believed that mycobacterial virulence is, in part, governed by several secreted virulence factors, such as members of the ESAT-6 protein family encoded by the RD1 genome region. Notably, the RD1 region is present in all virulent *M. tuberculosis* and *M. bovis* strains and is one of several regions of difference deleted from the genome of all strains of attenuated BCG (36). The presence or absence of these virulence factors are thought to result in differential host innate immune responses to virulent and attenuated mycobacteria that can be identified through the analysis of host gene expression data (37–39). In the present study, we have analyzed the global differential bovine macrophage transcriptional response to virulent *M. bovis* and attenuated BCG across an *in vitro* infection time course of 2, 6, and 24 h. Furthermore, we have applied a systems biology approach to reconstruct the complex molecular interactions and networks that illustrate the differential macrophage responses to these mycobacterial strains. Further work will be necessary to provide insights into whether key genes identified in this study represent aspects of attenuation regulated by the RD1 region.

Comparison of the MDM response to infection with *M. bovis* relative to BCG revealed the largest number of DE genes at 24 h post-infection (2,674 DE genes), which was 3.8- and 2.7-fold higher than the number of DE genes at the observed at 2 h (702 DE genes) and 6 h time points (1,000 DE genes), respectively (Figure 1). Notably, comparison between *M. bovis*- and BCG-infected MDM at the 2-h time point did not reveal significant differences in the expression profiles of key immune-related genes known to be important during mycobacterial infection. For example, mRNA transcripts were detected for genes encoding macrophage pattern recognition receptors (PRRs) such as *TLR2* and *TLR4*, which are involved in the recognition of evolutionarily conserved pathogen-associated molecular patterns (PAMPs), and inflammatory cytokines including *IL1B, IL6*, and *IL10* following infection; however, these were not DE between treatments (adjusted *P*-value of 0.01).

Systems analysis of the DE genes identified at the 24-h time point generated a biological interaction network that displayed both scale-free and small-world properties (Figure 3). In a scale-free network, nodes are highly clustered and can be connected to each other by relatively few steps due, in part, to the presence of a small number of highly connected hub nodes (34, 40). Hub nodes, identified as those nodes that have the highest DOC within a network, enable scale-free networks to be highly robust. The majority of nodes in the network are non-hub nodes with only a small number of connections; therefore, scale-free networks can withstand the removal of non-hub nodes and still retain the capacity to transmit information across the whole network. As such, these networks are said to be “error tolerant.” There are many examples of both small-world and scale-free networks, including metabolic networks (41–43), neural networks in the brain (44, 45), and the Internet (46). However, scale-free networks are vulnerable: should key hub nodes be removed, the network will be seriously compromised. Therefore, although scale-free and small-world networks are highly robust against random removal of non-hub nodes, they are vulnerable to targeted attacks on key hub nodes (47).

In the current study, *IKBKE, MYC*, and *NFKB1* were the top three ranking hub genes within the 24-h network (Figure 2). The importance of these genes is highlighted by the effect they have on the network based on the number of DE genes that are close neighbors (up to four edges away, Figure 4). There is not enough information available from the interaction databases to infer directionality for all interactions in the network, but the type of products associated with these hubs (inhibitors and activators, see below) suggest that most of their interactors lie downstream within a signaling cascade. The fact that nearly all of the DE genes in the network are linked by a maximum of four edges to one of the three main hub genes emphasizes the rapid and extensive response that can be initiated by change of expression in a very small number of genes. Furthermore, although the first- and second-degree DE neighbors of the hubs are mostly separate sub-groups, these sets of genes merge quickly into one large group after the next two extensions of the network. In other words, the overall number of DE genes (but not necessarily the type of expression change) could potentially be derived from changes at any one of the hubs, which points toward a single response captured by the network, compared to a group of separate responses. This correlates well with the detection of one common category (*Cell death*) found to be significantly over-represented in the GO analysis; however, observable differences in macrophage viability or morphology between BCG and *M. bovis* infections were not examined. The identification of three hubs instead of just one could be indicative of (i) redundancy, which has evolved for more robustness in the response to infections; or (ii) fine-tuning derived from interplay between different regulators, which could provide greater control of the response.

The products of the *IKBKE* and *NFKB1* genes are central to the NF-κB signaling pathway that regulates the expression of many immune-related genes following infection. *IKBKE* encodes a subunit of the inhibitor of κB (IκB) kinase (IKK) complex, which phosphorylates the NF-κB inhibitors (IKBs), resulting in the release of the NF-κB transcription factor complex, a subunit of which is encoded by the *NFKB1* gene (48). Notably, at the 24-h time point, both *IKBKE* and *NFKB1* were up-regulated in the *M. bovis*-infected MDM relative to BCG-infected MDM, suggesting that there is increased activation of NF-κB signaling following infection with the virulent mycobacterial strain. This is further supported by the increased relative expression of several NF-κB-inducible genes known to play a role in the host response to *M. bovis*, such as *IL1A, IL1B, IL6*, and *TNF* (49–51).

The up-regulation of the *IKBKE* and *NFKB1* hub genes indicates that activation of NF-κB and consequent downstream signaling and interactors of these hub genes (Figure 5) are crucial for the differential success of virulent and attenuated *M. bovis* strains within the host macrophage. Indeed, several pathogens, including *Helicobacter pylori* and *Chlamydia pneumoniae*, have been shown to manipulate the host NF-κB signaling pathway to ensure their survival (52). Evidence of the modulation of monocyte NF-κB signaling following mycobacterial infection has also been demonstrated. Dhiman et al. (53) showed that human monocytes infected with virulent *M. tuberculosis* exhibited increased NF-κB activity that resulted in up-regulation of NF-κB-inducible anti-apoptotic genes and increased survival relative to monocytes infected with avirulent *M. tuberculosis*. In the current study, we also observed increased expression of several NF-κB-inducible anti-apoptotic genes including *BIRC3, CFLAR*, and *TRAF2*, which were among the downstream interactors of the *IKBKE* and *NFKB1* hub genes (Figure 5). Furthermore, GO analysis of all nodes in the 24-h network revealed enrichment for genes involved in apoptosis, which is increasingly regarded as a host innate immune mechanism that contains and limits mycobacterial growth following infection (54–56). However, the induction of anti-apoptotic genes may represent an immuno-modulation strategy employed by the pathogen that inhibits apoptosis, enabling survival, and replication within the macrophage (55). Therefore, it is possible that virulent *M. bovis* targets key members of the NF-kB pathway causing the induction of anti-apoptotic mechanisms that result in prolonged viability within the bovine macrophage.

*MYC*, another highly connected hub gene identified within the 24-h network, displayed the same DOC as *IKBKE*. *MYC* encodes a multi-functional transcription factor that serves as a key regulator of cell proliferation and apoptosis and has been shown to suppress or activate the expression of its target genes (57, 58). A recent study has shown that *MYC* expression was induced in primary human blood-derived macrophages following *in vitro* infection with pathogenic and non-pathogenic mycobacterial species. Moreover, it was demonstrated that the MYC transcription factor mediated the suppression of intra-macrophage mycobacterial growth via the activation of cytokines including TNF and IL-6 (59). In the current study, *MYC* was down-regulated in *M. bovis*-infected MDM relative to BCG-infected MDM within the 24-h network. Furthermore, 51 of 73 (69.8%) of the downstream *MYC* interactors at the 24-h time point were also down-regulated (Figure 5). Previous work by our group using the same *M. bovis*-infected MDM described here demonstrated that *MYC* was down-regulated relative to non-infected MDM from the same animals (20, 22). It is also important to note that *MYC* was not DE in the BCG-infected MDM analyzed here relative to the same control MDM (unpublished results). Collectively, these results suggest that suppression of *MYC* following *M. bovis* infection results in the suppression of host pro-inflammatory responses leading to intracellular microbial survival and growth. Hence, *MYC* may serve as a key target for mycobacterial modulation of innate immune mechanisms, facilitating persistence within host macrophages.

Bottleneck nodes in the 24-h network were identified as nodes with a high BCI (Figure S1 in Supplementary Material). BCI has been shown to be associated with constrained evolutionary rates for gene or protein nodes in biological networks; studies involving eukaryote protein-interaction networks have demonstrated that bottleneck nodes evolve more slowly than non-bottleneck nodes (60, 61). Analyses of protein-interaction networks have also shown that bottleneck nodes are more likely to be essential for organismal survival than non-bottleneck nodes (62, 63).

In the current study, *EGR1*, together with *IKBKE* and *MYC*, was identified as a top bottleneck node within the 24-h interaction network (Figure S1 in Supplementary Material), with the majority of significantly DE genes interacting with *EGR1* showing changes at this time point (55 out of 85, Figure 5). *EGR1* encodes a zinc-finger transcription factor that regulates the expression of a large number of genes involved in cellular differentiation and mitogenesis. In addition, EGR-1 has also been shown to regulate the transcription of pro-inflammatory cytokines in murine macrophages in response to stimulation with bacterial antigens, including TNF and IL-6 (64, 65). To our knowledge, only one publication exists associating *EGR1* with mycobacteria–macrophage interactions (66), although it has recently been identified as part of a conserved macrophage core transcriptional response module that is DE in response to intracellular bacterial pathogens (67). Here, identification of *EGR1* as a key bottleneck gene suggests that this gene is a component of a differential bovine macrophage response module to virulent and attenuated mycobacterial strains.

It has been shown that many of the species and subspecies in the *M. tuberculosis* complex exhibit specific host association (68). It is believed that this host specificity is an evolutionary consequence of reciprocal adaptive genetic changes that have occurred due to strong selection pressures between the interacting pathogenic mycobacteria and their hosts (69, 70). Several examples of reciprocal traits involved in host-mycobacterial pathogen co-evolution have been described, including the ability of the host immune system to clear infection versus the ability of virulent pathogens to subvert and evade host immune responses (71). It is likely that the small-world properties of the 24-h network reflect host–pathogen co-evolution. Small-world networks are characterized by the presence of hub and bottleneck genes that interact with all other nodes within the network via a small number of steps. This model predicts that the expression and products of these hub and bottleneck genes enable the host to regulate an appropriate and efficient immune response to virulent or attenuated mycobacterial strains. Alternatively, key hub and bottleneck genes may serve as targets for the subversion of host immune responses by virulent pathogens that underlie successful infection, such as the suppression of apoptosis-related mechanisms by host transcription factors as demonstrated by Dyer et al. (72).

At the individual gene level, from the results presented here, it is not possible to determine whether host macrophage gene expression changes are due to regulation of host gene networks by the pathogen, the host, or a combination of both. In this regard, previous studies have demonstrated that pathogen-encoded protein virulence factors and, more recently, pathogen-encoded small regulatory RNA molecules manipulate and modulate host macrophage responses to facilitate intracellular survival and dissemination of pathogenic mycobacteria (73–79). Consequently, to fully understand the complex transcriptional regulatory interplay underlying host–mycobacterium interactions, it will be necessary to perform parallel high-throughput transcriptional profiling of mRNA and microRNA in both host and microbial cells (80, 81) and complement these with experimental detection of host–pathogen molecular interactions (82, 83).

## Conflict of Interest Statement

The Guest Associate Editor Kieran Meade declares that, despite having collaborated with the authors, Kate E. Killick, David A. Magee, Stephen D. E. Park, John A. Browne, Nicolas C. Nalpas, Eamonn Gormley, Stephen V. Gordon, David E. MacHugh, Karsten Hokamp the review process was handled objectively. The authors declare that the research was conducted in the absence of any commercial or financial relationships that could be construed as a potential conflict of interest.

## Supplementary Material

The Supplementary Material for this article can be found online at http://www.frontiersin.org/Journal/10.3389/fimmu.2014.00422/abstract

## Footnotes

^1^www.affymetrix.com

^2^www.bioconductor.org

^3^www.r-project.org

^4^www.bioconductor.org/packages/release/bioc/html/limma.html

^5^www.ensembl.org/biomart/martview

^6^www.innatedb.com

^7^www.cytoscape.org

^8^http://circos.ca

^9^http://igraph.sourceforge.net/doc/R/igraph.pdf
