# Supplementary material for: Key Hub and Bottleneck Genes Differentiate the Macrophage Response to Virulent and Attenuated Mycobacterium bovis
Source: Front Immunol. 2014 Oct 1;5:422. doi: 10.3389/fimmu.2014.00422 (PMC4181336; doi:10.3389/fimmu.2014.00422)
Supplement: Supplementary file 1 [file Presentation1.ZIP › Supp Table 1.DOCX]

**Table S1:** Real time qRT-PCR primers used for microarray validation

| **Gene name** | **Gene Symbol** | **Ensembl ID** | **Forward Primer Sequence (5′-3′)** | **Reverse Primer Sequence (5′-3′)** | **Amplicon size (bp)** | **Exon spanning** |
| --- | --- | --- | --- | --- | --- | --- |
| Chemokine (C-C motif) ligand 4 | *CCL4* | ENSBTAG00000025257 | AGCTGTGGTATTCCAGACCAA | TCAAGGTCATCCACGTACTCC | 87 | 2-3 |
| Chemokine (C-C motif) ligand 5 | *CCL5* | ENSBTAG00000007191 | AGCAGTTGTCTTTATCACCAGGA | TCCAAAGCGTTGATGTACTCTC | 87 | 2-3 |
| Chemokine (C-C motif) ligand 20 | *CCL20* | ENSBTAG00000021326 | AATTAGCTGTGTGTGCAGATCC | CATCCTTTTGACTCTTTGACTGA | 80 | 3-4 |
| CD40 molecule, TNF receptor superfamily member 5 | *CD40* | ENSBTAG00000020736 | TCCTGTTTGCTGTCCTGTTG | GATCCTGCCTTTCAGCCATA | 96 | 7-9 |
| Complement factor B | *CFB* | ENSBTAG00000007450 | CTTCATTCAAGTTGGCGTGA | GCAGCACCTGGTAGAGGTTG | 110 | 17-18 |
| Chemokine (C-X-C motif) ligand 2 | *CXCL2* | ENSBTAG00000037558 | TGGTCAGGAAGTGTGTCTCAA | TCAGTTGGCACTAGCCTTGTT | 85 | 3-4 |
| Interleukin 15 | *IL15* | ENSBTAG00000018200 | ACCATGCTAGCAAACAGCAA | TTCCTCCAGTTCCTCACATTC | 81 | 5-6 |
| Interleukin 1, beta | *IL1B* | ENSBTAG00000001321 | ACCTGAACCCATCAACGAAATG | TAGGGTCATCAGCCTCAAATAACA | 74 | 2-3 |
| Interleukin 6 | *IL6* | ENSBTAG00000014921 | ATCAGAACACTGATCCAGATCC | CAAGGTTTCTCAGGATGAGG | 145 | 4-5 |
| Interferon regulatory factor 1 | *IRF1* | ENSBTAG00000031231 | GCCCACCTCTGTCTATGGAG | CAGCTGGGATCCATGTTCTT | 111 | 9-10 |

| **Gene name** | **Gene Symbol** | **Ensembl ID** | **Forward Primer Sequence (5′-3′)** | **Reverse Primer Sequence (5′-3′)** | **Amplicon size (bp)** | **Exon spanning** |
| --- | --- | --- | --- | --- | --- | --- |
| Nuclear factor of kappa light polypeptide gene enhancer in B-cells 2 (p49/p100) | *NFKB2* | ENSBTAG00000006017 | CCTGCTGAATGCTCTGTCTG | TCCTCCTTCACCTCTGTGCT | 102 | 23-24 |
| Peptidylprolyl isomerase A (cyclophilin A) | *PPIA* | ENSBTAG00000012003 | CATACAGGTCCTGGCATCTTGTCC | CACGTGCTTGCCATCCAACC | 108 | 4-5 |
| Tumor necrosis factor (TNF superfamily, member 2) | *TNF* | ENSBTAG00000025471 | GCTCCAGAAGTTGCTTGTGC | AACCAGAGGGCTGTTGATGG | 149 | 1-2 |
