# Supplementary material for: Key Hub and Bottleneck Genes Differentiate the Macrophage Response to Virulent and Attenuated Mycobacterium bovis
Source: Front Immunol. 2014 Oct 1;5:422. doi: 10.3389/fimmu.2014.00422 (PMC4181336; doi:10.3389/fimmu.2014.00422)
Supplement: Supplementary file 1 [file Presentation1.ZIP › Supp Table 5.DOCX]

**Table S5:** Comparison of fold-changes in gene expression based on microarray and real time qRT-PCR results

| **Gene symbol** | **Gene description** | **2 hour mean fold-changes in gene expression** | | | **6 hour mean fold-changes in gene expression** | | | **24 hour mean fold-changes in gene expression** | | |
| --- | --- | --- | --- | --- | --- | --- | --- | --- | --- | --- |
|  |  | **Microarray data** | **Real time qRT-PCR** | **Adjusted *P*-value** | **Microarray data** | **Real time qRT-PCR** | **Adjusted *P*-value** | **Microarray data** | **Real time qRT-PCR** | **Adjusted *P*-value** |
| *CCL4* | A proinflammatory and chemotactic chemokine | Not DE | +5.82 | *P* ≤ 0.001 | +2.34 | +5.14 | *P* ≤ 0.01 | +2.17 | +4.36 | *P* ≤ 0.01 |
| *CCL5* | A proinflammatory chemokine involved in the chemotaxis of monocytes and T-helper cells | +4.27 | +7.44 | *P* ≤ 0.001 | +2.63 | +5.04 | *P* ≤ 0.001 | +2.49 | +5.35 | *P* ≤ 0.01 |
| *CCL20* | A chemokine involved in the chemoattraction of lymphocytes and neutrophils | +7.20 | +15.67 | *P* ≤ 0.001 | +11.23 | +23.89 | *P* ≤ 0.001 | +3.15 | +6.81 | *P* ≤ 0.01 |
| *CD40* | A member of the TNF-receptor superfamily; mediates the immune and inflammatory responses | +1.79 | +3.34 | *P* ≤ 0.001 | +1.51 | +2.61 | *P* ≤ 0.001 | +1.63 | +2.52 | *P* ≤ 0.05 |
| *CFB* | A component of the alternative pathway of complement activation | +3.65 | +5.61 | *P* ≤ 0.001 | +2.93 | +4.96 | *P* ≤ 0.001 | +3.14 | 6.33 | *P* ≤ 0.01 |
| *CXCL2* | An immunoregulatory chemokine produced by activated monocytes and neutrophils at sites of inflammation | Not DE | +1.50 | *P* ≤ 0.05 | +5.43 | +5.60 | *P* ≤ 0.001 | +4.47 | +5.68 | *P* ≤ 0.01 |
| *IL1B* | A cytokine that mediates the inflammatory response including cell proliferation, differentiation and apoptosis | +2.67 | +5.52 | *P* ≤ 0.001 | +6.35 | +13.60 | *P* ≤ 0.001 | +3.74 | +6.82 | *P* ≤ 0.01 |
| *IL6* | A cytokine that functions in inflammation and the maturation of B cells | +2.62 | +2.81 | *P* ≤ 0.05 | +3.59 | +6.59 | *P* ≤ 0.001 | +4.85 | +10.16 | *P* ≤ 0.01 |
| *IL15* | A cytokine that regulates T and natural killer cell activation and proliferation | +2.13 | +2.62 | *P* ≤ 0.001 | +2.82 | +3.38 | *P* ≤ 0.001 | Not DE | +1.57 | *P* ≤ 0.05 |
| *IRF1* | A member of the interferon regulatory transcription factor family; an activator of interferon alpha and beta transcription | Not DE | +2.31 | *P* ≤ 0.001 | +2.27 | +2.59 | *P* ≤ 0.01 | Not DE | +1.80 | *P* ≤ 0.05 |
| *NFKB2* | A pleiotropic transcription factor involved in inflammation, immunity, differentiation, cell growth and apoptosis | +1.79 | +1.77 | *P* ≤ 0.001 | +1.61 | +1.85 | *P* ≤ 0.05 | +2.04 | +2.56 | *P* ≤ 0.05 |
| *TNF* | A proinflammatory cytokine secreted by macrophages involved in the regulation of cell proliferation, differentiation and apoptosis | +1.98 | +2.35 | *P* ≤ 0.01 | +5.76 | +7.35 | *P* ≤ 0.001 | +2.71 | +3.89 | *P* ≤ 0.01 |

Geometric mean fold-changes of gene expression (M. bovis-infected relative to BCG-infected MDM) are given for the microarray and real time qRT-PCR data at each time point. For the microarray data, genes for which geometric mean values are provided were differentially expressed (adjusted *P*-value ≤ 0.05); ‘Not DE’ denotes genes that were not differentially expressed (adjusted *P*-value ≥ 0.05). Benjamini-Hochberg adjusted *P*-values are given for the real time qRT-PCR data. Descriptions of the function of each gene were obtained from the GeneCards version 3 database ([Safran *et al.* 2010](#_ENREF_9)).
