# Supplementary material for: Key Hub and Bottleneck Genes Differentiate the Macrophage Response to Virulent and Attenuated Mycobacterium bovis
Source: Front Immunol. 2014 Oct 1;5:422. doi: 10.3389/fimmu.2014.00422 (PMC4181336; doi:10.3389/fimmu.2014.00422)
Supplement: Supplementary file 1 [file Presentation1.ZIP › Supp Figures.PDF]

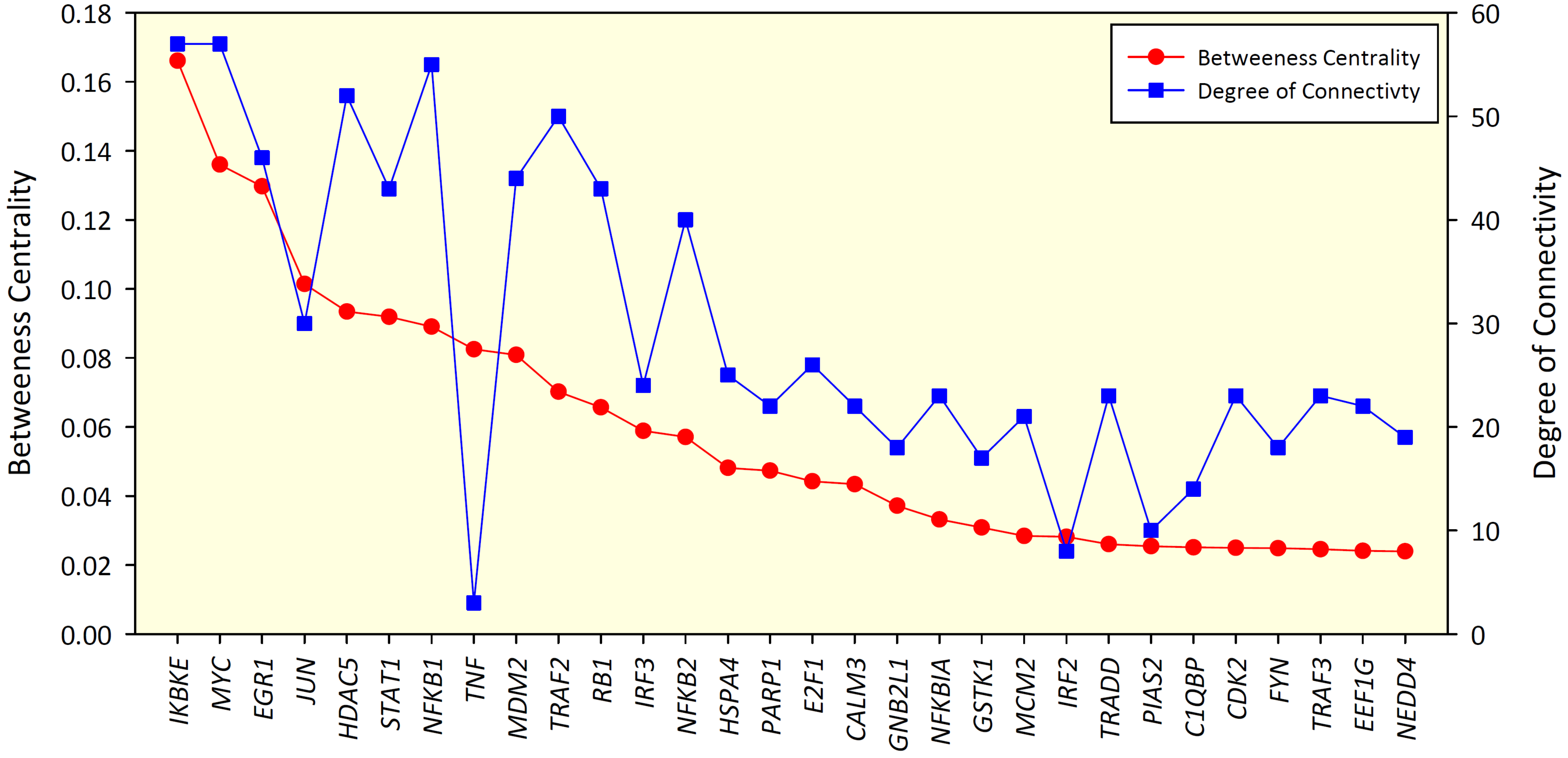

Figure S1: Top 30 nodes in the 24 h interaction network that displayed the highest BCI scores (red line). The DOC for these nodes is also shown (blue line).

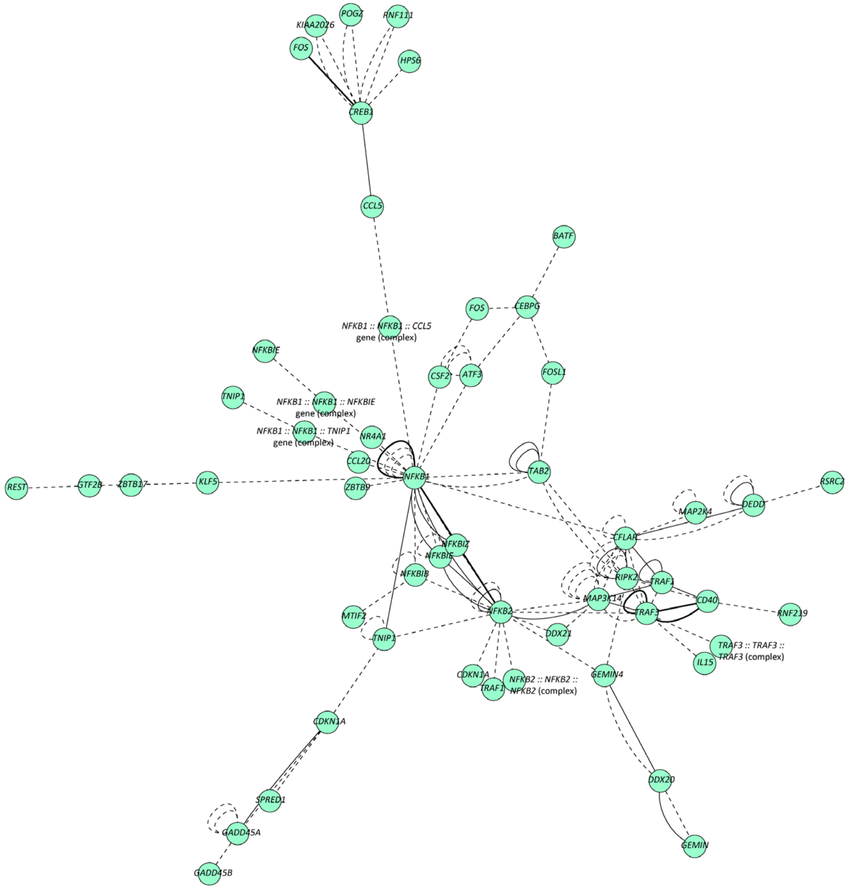

Figure S2: Interaction networks for the 2 h infection time point.

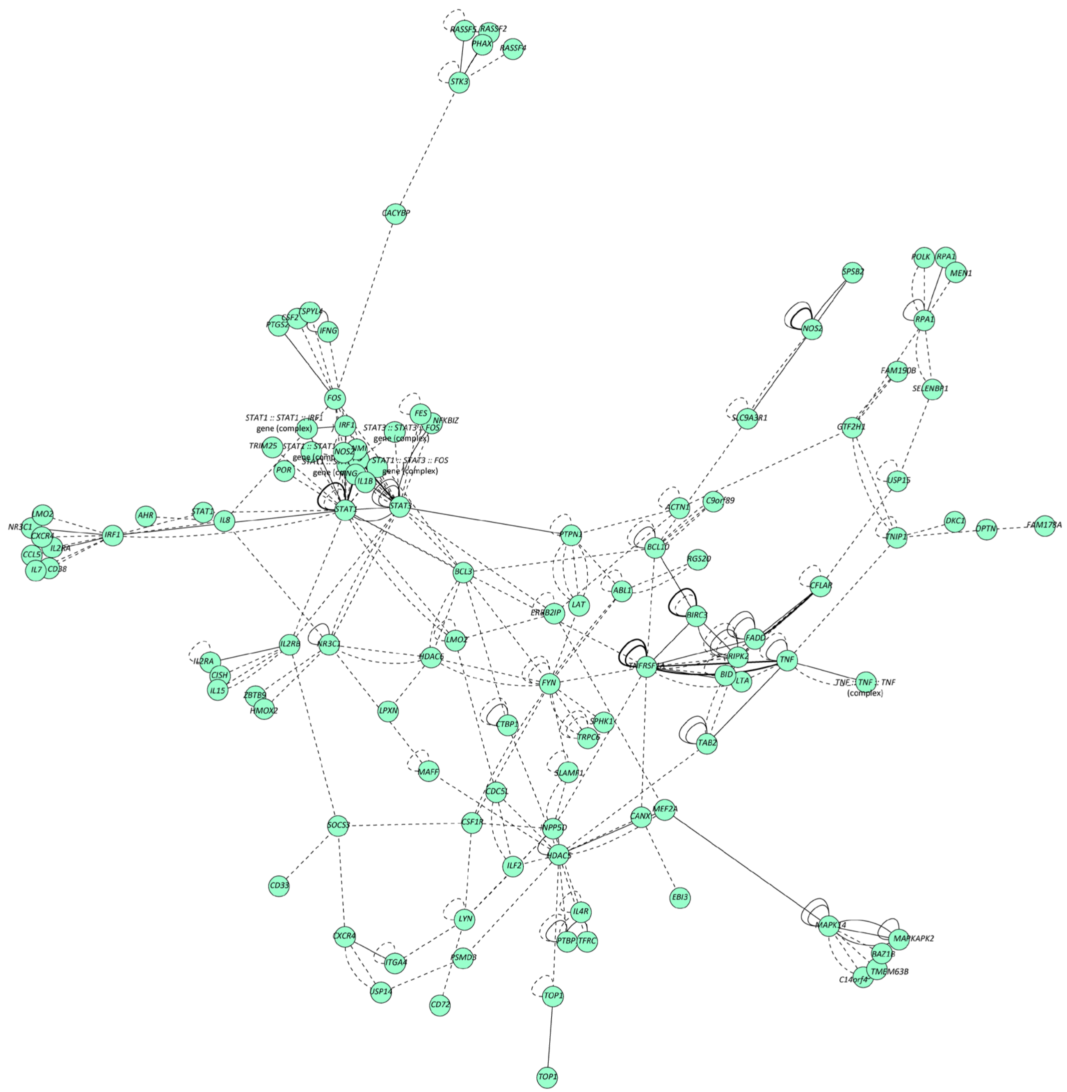

Figure S3: Interaction networks for the 6 h infection time point.

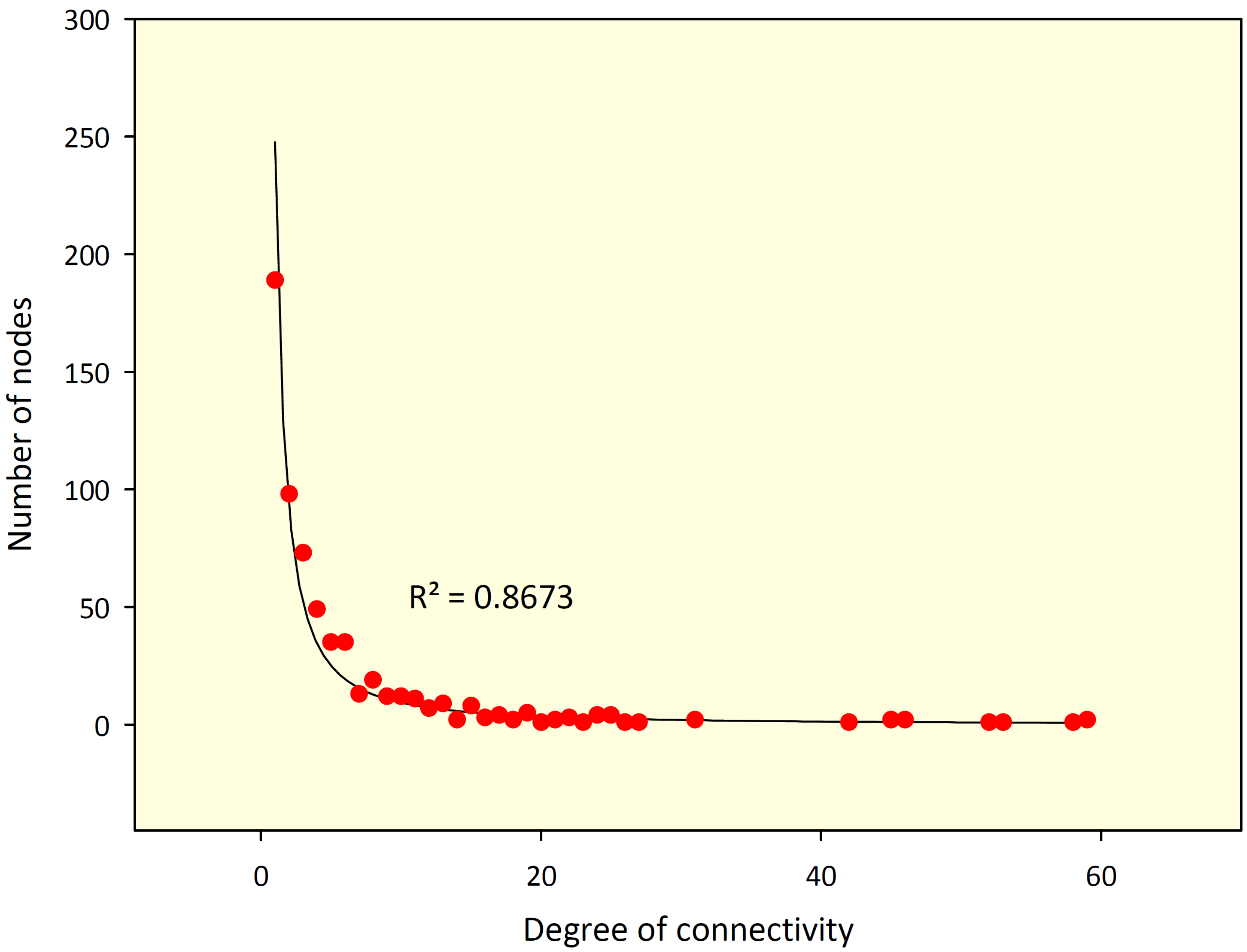

Figure S4: Power law distribution fitted to the node degree distribution of the 24 h network.
