# Supplementary material for: Key Hub and Bottleneck Genes Differentiate the Macrophage Response to Virulent and Attenuated Mycobacterium bovis
Source: Front Immunol. 2014 Oct 1;5:422. doi: 10.3389/fimmu.2014.00422 (PMC4181336; doi:10.3389/fimmu.2014.00422)
Supplement: Supplementary file 1 [file Presentation1.ZIP › Supp Material & captions.DOCX]

***Supplementary Material***

**Key hub and bottleneck genes differentiate the macrophage response to virulent and attenuated *Mycobacterium bovis***

**Kate E. Killick^1,2^*, David A. Magee^1^, Stephen D. E. Park^1,3^, Maria Taraktsoglou^1,4^, John A. Browne^1^, Kevin M. Conlon^5,6^, Nicolas C. Nalpas^1^, Eamonn Gormley^7^, Stephen V. Gordon^5,8^, David E. MacHugh^1,8^, Karsten Hokamp^9^**

^1^Animal Genomics Laboratory, UCD School of Agriculture and Food Science, University College Dublin, Dublin 4, Ireland

^2^Systems Biology Ireland, UCD Conway Institute of Biomolecular and Biomedical Research, University College Dublin, Dublin 4, Ireland

^3^IdentiGEN Ltd., Trinity Enterprise Centre, Pearse Street, Dublin 2, Ireland

^4^Biological Agents Unit, Health and Safety Executive, Leeds, LS11 9AT, United Kingdom

^5^UCD School of Veterinary Medicine, University College Dublin, Dublin 4, Ireland

^6^Science Foundation Ireland (SFI), Wilton Park House, Wilton Place, Dublin 4, Ireland

^7^Tuberculosis Diagnostics and Immunology Research Centre, UCD School of Veterinary Medicine, University College Dublin, Ireland

^8^UCD Conway Institute of Biomolecular and Biomedical Research, University College Dublin, Dublin 4, Ireland

^9^Smurfit Institute of Genetics, Trinity College, Dublin 2, Ireland

*** Correspondence:** Kate E. Killick, Systems Biology Ireland, UCD Conway Institute of Biomolecular and Biomedical Research, University College Dublin, Dublin 4, Ireland.

[kate.killick@ucd.ie](mailto:kate.killick@ucd.ie)

1. **Real time quantitative reverse transcription (qRT)-PCR validation of microarray results**

cDNA for real time qRT-PCR analysis was prepared from 80 ng of total RNA isolated from the same MDM samples used for microarray analysis using a High Capacity cDNA Reverse Transcription Kit (Applied Biosystems^®^, Warrington, UK). cDNA conversions were performed in 20 µl reaction using random primers as per the manufacturer’s instructions. In addition, 80 ng of pooled RNA (~10 RNA samples per pool) was included in non-reverse transcriptase (non-RT) control reactions to test for the presence of contaminating genomic DNA during real-time qRT-PCR analysis. All cDNA samples and non-RT controls were diluted 1:8 using RNAse- and DNAse-free water and stored at -20°C prior to real time qRT-PCR analysis.

For real time qRT-PCR analysis, intron-spanning primers were designed for a panel of 12 immune genes using the Primer3Plus package (Untergasser et al., 2007) and commercially-synthesised (Eurofins MWG Operon, Ebersberg, Germany). Selection of these genes was based on the documented biological role that these genes play in the immune response to mycobacterial infection (van Crevel et al., 2002; Cooper et al., 2011). These genes were: *CCL20*, *CCL4*, *CCL5*, *CD40*, *CFB*, *CXCL2*, *IL15*, *IRF1*, *NFKB2*, *IL1B*, *IL6*, *TNFA*.

Supplementary **Table S1** provides sequence information for all primer pairs used. Real time qRT-PCR reactions (20 µl final volume) were performed on 96-well plates using Fast SYBR^®^ Green Master Mix (Applied Biosystems^®^, Life Technologies Corporation, Warrington, UK) on a 7500 Fast Real Time PCR System (Applied Biosystems^®^) as per manufacturer’s instructions. Real time qRT-PCR amplifications contained 2 µl of the diluted cDNA (equivalent to 1.0 ng of total RNA). A final concentration of 300 nM of each forward and reverse primer was included in each amplification reaction. Non-template real time qRT-PCR controls and a seven-point standard curve prepared from 1:2 serial dilutions of pooled conventionally-prepared cDNA *M. bovis*-challenged MDM sample were included on every real time qRT-PCR plate. PCR thermal cycling conditions for each amplicon comprised one cycle at 50°C for 2 minutes, one cycle at 95°C for 20 seconds, followed by 40 cycles at 95°C for 3 seconds and 60°C for 30 seconds. A dissociation step was included for all amplifications to confirm the presence of single discrete PCR products of the expected size; this was further confirmed by visualisation of the amplification products on 2% agarose gels stained with 0.5 mg/ml ethidium bromide (Invitrogen™, Life Technologies Corporation, Paisley, UK).

Sanger sequencing was also performed using PCR amplicons generated from cDNA and the real time qRT-PCR primers listed in supplementary **Table S1** to confirm the identity of the genes targeted by the real time qRT-PCR primers. For this, conventional PCR amplifications were prepared using 2 µl of cDNA from the top standard of the standard curve in 30 µl reactions containing 1× Platinum Taq DNA Polymerase buffer (Invitrogen™), 200 nM each dNTP, 0.2 µM forward primer, 0.2 µM reverse primer, 2.0-2.5 mM MgCl2 and 0.2 units Platinum Taq polymerase (Invitrogen™). Conventional PCR was performed using 5 min initial denaturation at 95°C, followed by 40 cycles denaturation (95°C, 45 s), annealing (54°C-61°C, 45 s); and extension (72°C, 1 min), with a final extension (72°C, 10 min). Sanger sequencing was performed commercially (Source Bioscience Ltd., Dublin, Ireland) using the forward real time qRT-PCR primers and yielded PHRED quality scores ≥ 20. PCR amplicon sequences were subsequently aligned to sequences in the *B. taurus* RefSeq database ([www.ncbi.nlm.nih.gov](http://www.ncbi.nlm.nih.gov)) to confirm the identity of the genes targeted by the real time qRT-PCR primers. All sequences aligned correctly to all 12 genes included for real time qRT-PCR analysis.

## Statistical analysis of real time qRT-PCR data

All real time qRT-PCR data, including primer efficiency estimations using the standard curves, were analysed using the qbase^PLUS^ software package (Hellemans et al., 2007) [Biogazelle NV, Zwijnaarde, Belgium]. Real time qRT-PCR efficiency correction and normalisation was performed using the peptidylprolyl isomerase A (cyclophilin A) gene (*PPIA*) as a reference gene based on GeNorm analysis performed by us in a previous study involving bovine MDM (Taraktsoglou et al., 2011).

Calibrated normalised relative quantities (CNRQ) of gene expression for each analysed sample, as generated by the qbase^PLUS^ package, were used to calculate fold-changes in expression for each gene. For this, the CNRQ value generated for each *M. bovis*- and BCG-infected MDM sample was log_2_-transformed. Log_2_ fold-changes in gene expression were calculated by subtracting the log_2_ CNRQ values for the BCG-infected MDM from the log_2_ CNRQ values for the *M. bovis*-infected MDM from the corresponding animal at the corresponding time point. All statistical analyses for real time qRT-PCR analyses were performed using the SPSS statistical package (IBM Corporation, Armonk, NY, USA) and the Minitab version 16 statistical package (Minitab Ltd., Coventry, UK).

Kolmogorov-Smirnov tests of normality were applied to log_2_ fold-change differences in gene expression at each time point prior to statistical analysis to ensure the data conformed to a normal distribution. Q-Q plots of log_2_ fold-change differences in gene expression at each time point were also constructed and visually inspected. For normally distributed data (Kolmogorov-Smirnov test *P* values ≥ 0.05), two-tailed paired Student’s *t*-tests were used to assess significant differences in mean log_2_ fold-changes in gene expression between the *M. bovis*-infected MDM relative to BCG-infected MDM at each time point. For non-normally distributed data (Kolmogorov-Smirnov test *P* values ≤ 0.05), Wilcoxon signed-rank tests were performed to test for differences in the medians of the log_2_ CNRQ values for the *M. bovis*- and BCG-infected MDM. fold-changes in gene expression between the *M. bovis*-infected MDM relative to BCG-infected MDM at each time point. The Benjamini-Hochberg multiple-testing correction method (Benjamini and Hochberg, 1995) was applied to the raw *P*-values generated for each gene analysed by real time qRT-PCR using the stats package in R.

Geometric mean fold-changes in expression were generated for each gene by back-transformation of the mean log_2_ fold-changes in gene expression of the *M. bovis*-infected MDM relative to BCG-infected MDM at each time point. Genes displaying an adjusted *P*-value ≤ 0.05 were regarded as being differentially expressed based on real time qRT-PCR data. For comparative purposes, an adjusted *P*-value threshold ≤ 0.05 was used to identify differentially expressed genes from the microarray data.

## Real time quantitative reverse transcription PCR analysis and validation of microarray results

The relative fold-changes in expression between the *M. bovis*- and BCG-infected MDM as determined through real time qRT-PCR analysis and microarray analyses for the panel of 12 genes listed above are provided in supplementary **Table S5**. The direction in expression of all 12 genes were the same on both the microarray and real time qRT-PCR platforms, with all genes displaying upregulation in the *M. bovis*-infected MDM relative to the BCG-infected MDM. All genes were differentially expressed across all time points based on the real time qRT-PCR results (adjusted *P*-value ≤ 0.05), while three genes (*CCL4*, *CXCL2* and *IRF1*) and two genes (*IL15* and *IRF1*) were not differentially expressed at 2 h and 24 h, respectively, based on microarray data (adjusted *P*-value ≥ 0.05). Of the total 36 pairwise comparisons between the microarray and the real time qRT-PCR data (*i.e.* 12 genes across three time points), 31 were significant across both platforms (adjusted *P*-value ≥ 0.05), with the same direction (*i.e.* upregulated) of gene expression observed for both platforms. This yielded a concordance rate of 86.11% for both platforms. This concordance is similar to that observed by us in previous transcriptomic studies of the host response to mycobacterial infection using the Affymetrix^®^ GeneChip^®^ Bovine Genome Array (Killick et al., 2011; MacHugh et al., 2012; Magee et al., 2012). The discrepancies between the microarray and real time qRT-PCR data for the three genes at the 2 h time point and two genes at the 24 h time point may reflect differences in the sensitivity of the two analytical methods used and/or differences in the mRNA transcripts targeted by the probes (microarray) and primer pairs (real time qRT-PCR) used for the two forms of gene expression analysis (Draghici et al., 2006; Morey et al., 2006).

1. **Supplementary Figures and Tables**

**Figure S1:** Top 30 nodes in the 24 h interaction network that displayed the highest BCI scores (red line). The DOC for these nodes is also shown (blue line). [**Supp_Figure_S1.tif**]

**Figure S2:** Interaction networks for the 2 h infection time point. [**Supp_Figure_S2.tif**]

**Figure S3:** Interaction networks for the 6 h infection time point. [**Supp_Figure_S3.tif**]

**Figure S4**: Power law distribution fitted to the node degree distribution of the 24 h network. [**Supp_Figure_S4.tif**]

**Table S1:** Real time qRT-PCR primers used for microarray validation. [**Supp_Table_S1.docx**]

**Table S2:** DE genes detected between a paired comparison of virulent *M. bovis* versus attenuated BCG MDM infections at 2 h post infection, after filtering for an adjusted *P*-value of ≤ 0.01. [**Supp_Table_S2.xlsx**]

**Table S3:** DE genes detected between a paired comparison of virulent *M. bovis* versus attenuated BCG MDM infections at 6 h post infection, after filtering for an adjusted *P*-value of ≤ 0.01. [**Supp_Table_S3.xlsx**]

**Table S4:** DE genes detected between a paired comparison of virulent *M. bovis* versus attenuated BCG MDM infections at 24 h post infection, after filtering for an adjusted *P*-value of ≤ 0.01. [**Supp_Table_S4.xlsx**]

**Table S5**: Real time qRT-PCR validation of microarray results. [**Supp_Table_S1.docx**]

1. **References**

Benjamini, Y, and Hochberg, Y (1995). Controlling the false discovery rate - a practical and powerful approach to multiple testing. *Journal of the Royal Statistical Society Series B-Methodological* 57**,** 289-300.

Cooper, AM, Mayer-Barber, KD, and Sher, A (2011). Role of innate cytokines in mycobacterial infection. *Mucosal Immunol* 4**,** 252–260.

Draghici, S, Khatri, P, Eklund, AC, and Szallasi, Z (2006). Reliability and reproducibility issues in DNA microarray measurements. *Trends Genet* 22**,** 101-109. doi: 10.1016/j.tig.2005.12.005.

Hellemans, J, Mortier, G, De Paepe, A, Speleman, F, and Vandesompele, J (2007). qBase relative quantification framework and software for management and automated analysis of real-time quantitative PCR data. *Genome Biol* 8**,** R19. doi: gb-2007-8-2-r19 [pii]

10.1186/gb-2007-8-2-r19.

Killick, KE, Browne, JA, Park, SD, Magee, DA, Martin, I, Meade, KG, Gordon, SV, Gormley, E, O'Farrelly, C, Hokamp, K, and MacHugh, DE (2011). Genome-wide transcriptional profiling of peripheral blood leukocytes from cattle infected with *Mycobacterium bovis* reveals suppression of host immune genes. *BMC Genomics* 12**,** 611. doi: 10.1186/1471-2164-12-611.

MacHugh, DE, Taraktsoglou, M, Killick, KE, Nalpas, NC, Browne, JA, Park, SD, Hokamp, K, Gormley, E, and Magee, DA (2012). Pan-genomic analysis of bovine monocyte-derived macrophage gene expression in response to *in vitro* infection with *Mycobacterium avium* subspecies *paratuberculosis*. *Vet Res* 43**,** 25. doi: 10.1186/1297-9716-43-25.

Magee, DA, Taraktsoglou, M, Killick, KE, Nalpas, NC, Browne, JA, Park, SD, Conlon, KM, Lynn, DJ, Hokamp, K, Gordon, SV, Gormley, E, and MacHugh, DE (2012). Global gene expression and systems biology analysis of bovine monocyte-derived macrophages in response to *in vitro* challenge with *Mycobacterium bovis*. *PLoS ONE* 7**,** e32034. doi: 10.1371/journal.pone.0032034.

Morey, JS, Ryan, JC, and Van Dolah, FM (2006). Microarray validation: factors influencing correlation between oligonucleotide microarrays and real-time PCR. *Biol Proced Online* 8**,** 175-193. doi: 10.1251/bpo126.

Taraktsoglou, M, Szalabska, U, Magee, DA, Browne, JA, Sweeney, T, Gormley, E, and MacHugh, DE (2011). Transcriptional profiling of immune genes in bovine monocyte-derived macrophages exposed to bacterial antigens. *Vet Immunol Immunopathol* 140**,** 130-139. doi: 10.1016/j.vetimm.2010.12.002.

Untergasser, A, Nijveen, H, Rao, X, Bisseling, T, Geurts, R, and Leunissen, JA (2007). Primer3Plus, an enhanced web interface to Primer3. *Nucleic Acids Res* 35**,** W71-74. doi: 10.1093/nar/gkm306.

van Crevel, R, Ottenhoff, TH, and van der Meer, JW (2002). Innate immunity to *Mycobacterium tuberculosis*. *Clin Microbiol Rev* 15**,** 294-309.
